# Lessons Learned by Collaborating with Structurally Vulnerable Veterans via a Veterans Engagement Group

**DOI:** 10.1007/s11606-021-07075-y

**Published:** 2022-03-29

**Authors:** Erica Hua Fletcher, Sonya Gabrielian, Lidia Brown, Juan Carlos Gough, Roya Ijadi-Maghsoodi, Ippolytos Kalofonos, Mariam Nazinyan, Erika Orellana, Kenneth Wells

**Affiliations:** 1 UCLA Jane and Terry Semel Institute for Neuroscience and Human Behavior, Center for Health Services and Society, Los Angeles, USA; 2 UCLA/VA Center of Excellence on Veteran Resilience and Recovery, Los Angeles, CA USA; 3 Desert Pacific Mental Illness Research, Education, and Clinical Center (MIRECC), VA Greater Los Angeles Healthcare System, Los Angeles, USA; 4 Health Services Research & Development (HSR&D), Center for the Study of Healthcare Innovation, Implementation, & Policy, VA Greater Los Angeles Healthcare System Health Services, Los Angeles, USA; 5 Department of Psychiatry and Biobehavioral Sciences, UCLA David Geffen School of Medicine, Los Angeles, USA; 6 UCLA Center for Social Medicine and Humanities, Los Angeles, USA; 7 UCLA Fielding School of Public Health, Los Angeles, USA

## INTRODUCTION

Community-engaged research methodologies have been promoted recently, as stakeholder participation in research has been shown to improve the relevance, rigor, and acceptance of research findings across diverse populations.^1,2^ Such methodologies value stakeholder trust, mutual knowledge exchange, and coequal participation between researchers and stakeholders.^1^ The 2010 Patient Protection and Affordable Care Act supported this paradigm shift by creating the Patient-Centered Outcomes Research Institute, which provides grant funding, training, and technical support to researchers committed to stakeholder engagement.^3^ Likewise, over the last decade, VA Office of Research Protections, Policy, and Education and Health Services Research and Development Services, along with their Centers of Innovation for Veteran-Centered and Value-Driven Care, have informed research priorities and practices via a National Workgroup on Veteran Engagement and publication of a guide to establish Veteran Engagement Groups (VEGs).^4–6^ Drawing from principles of community-engaged research, VEGs serve to “systematically and consistently incorporate Veteran feedback” into studies at VA research centers; VEG meetings are characterized as settings in which “Veterans review research projects or proposals at various stages and dialogue with other stakeholders to provide individual feedback to researchers.”^4^

Of Veterans, 10.2% experience homelessness as adults with high rates of illness and substance use and with 35% reporting use of ED services in 2019.^7,8^ Incorporating this population into research processes is essential to promote social equity and to improve research related to Veterans’ health, housing, and services..^9^ The UCLA/VA Center of Excellence (COE) on Veteran Resilience and Recovery developed a VEG that is unique in recruiting homeless-experienced Veterans with behavioral health issues, to solicit guidance about studies funded by the COE or conducted by affiliated investigators.^10^ This paper describes the VEG’s development, operation, and impact; considers best practices for stakeholder engagement; and discusses challenges to shared leadership in research. It is co-authored with two Veterans who are involved in recruitment and retention of Veterans to the VEG.

## UCLA/VA COE VEG PROGRAM DESCRIPTION

The COE was established in 2018 to improve resilience and recovery of Veterans with behavioral health challenges (i.e., mental illness, substance use disorders) through research and provider training activities at VA of Greater Los Angeles (VAGLA).^10^ The mission of the COE’s Research Division includes a focus on stakeholder engagement, with dedicated funds for the VEG’s formation and operation.^1,2^

VEG participants reflect the demographics of Veterans served at VAGLA. Of the 10 Veterans who have served on the VEG, 7 were men (including 1 female-to-male trans man) between the ages of 30 and 68, with an average age of 45; 3 were women between the ages of 39 and 68, with an average age of 53. Four identified as Black, 4 as White, 1 as multi-racial, and 1 undisclosed. For comparison, in FY 2019, VAGLA provided services to 16,000 homeless-experienced Veterans, 9% were women with an average age of 47, and 91% were men with an average age of 57.^11^ Most VEG participants used VA homeless services and have a history of behavioral health challenges; many suffer from serious physical ailments, such as chronic pain.

Veterans are recruited to the VEG via word of mouth and recommendations from homeless program clinicians. Participation can be a one-time event (with an open invitation to attend additional meetings) or longitudinal. VEG meetings were initially held 6–10 times a year, with 3–5 present at a VEG meeting, ≥2 COE investigators, and 2 COE staff. To encourage participation, COE staff provide frequent meeting reminders, often contacting Veterans 3–5 times to ensure their attendance and providing transportation via bus vouchers. Veterans receive a $50 gift card for their counsel, an amount higher than the $5–20 per hour typical for participation in research studies.

## MEETING PROCEEDINGS AND ADAPTATION

The COE’s leadership worked with Veterans to develop the VEG’s process: VEG facilitators gave Veterans an orientation and a meeting outline, and explicitly encouraged all viewpoints and questions. Veterans served as members of the pilot study review team and in a consulting role for researchers with COE funding. Investigators seeking COE funding for pilot research projects were required to present proposals to the VEG and incorporate Veterans’ feedback. At the conclusion of these studies, researchers provided findings to the VEG to assist with the dissemination of findings. This process created a feedback loop between Veterans and researchers: Veterans saw how their feedback shaped the research process and the study outcomes, and researchers utilized their feedback to adapt and align projects to fit the needs of the Veteran community.^10^

Investigators modified meeting proceedings based on participating Veterans’ recommendations. During the first year of operation, researchers struggled to translate study proposals to lay audiences, while Veterans were not familiar with the traditional academic model of assessing the merits of an approach during the review process.^10^ Thus, facilitators changed the process to more fully orient researchers to the VEG’s mission and goals and to encourage them to solicit specific feedback on aspects of the presented study. They also created a less structured format allowing Veterans to openly discuss their thoughts on each proposal. Some Veterans adopted informal facilitator roles to ask questions of researchers to translate aspects of their proposals to fellow Veterans, when they seemed unsure of what was being discussed. This modified approach enabled differing forms of expertise to be recognized.

## IMPACT

Since the VEG’s creation in 2018, Veterans have advised VA researchers and clinicians on a range of topics, from feedback on a pilot intervention for Veterans who hear voices to a family resilience program for homelessness-experienced families. In total, they have consulted on 6 proposed studies. Veterans noted helping fellow Veterans and improving systems of care as motivations for participation.^4^ They reported a sense of camaraderie in sharing their challenges accessing VA services and validation in knowing that researchers wanted to incorporate their perspectives. For these reasons, some Veterans felt the meetings served as a therapeutic space for them to “bring their full selves to the table.” Despite these successes, over 2 years, 4 Veterans stopped attending due to loss of contact, moving residences, or incompatible work schedules.

## LESSONS PARTNERING WITH STRUCTURALLY VULNERABLE VETERANS

Homeless-experienced Veterans with behavioral health needs experience more structural vulnerability and discrimination than middle-class Veterans who often comprise VEGs.^7^ Researchers working with structurally vulnerable Veterans must be attentive to ethical issues related to economic exploitation and systemic racism, alongside other forms of discrimination.^12^ They must also pay special attention to power dynamics associated with compensation, homelessness, and communication, including Veterans’ understanding of research issues and language.^4^

## BENEFITS

COE investigators understand Veterans’ contributions as work and believe that compensation demonstrates respect for participants’ time,^13,14^ and that the amount should be similar to other research centers at UCLA and VAGLA. Likewise, Veterans, particularly those living in VA transitional housing, report that compensation is fair and a lifeline for them, enabling them to purchase necessities. Some suggest that increasing the amount, especially for those with children, may incentivize long-term participation of a diverse group of Veterans. Veterans note the hospitality felt while sharing refreshments in meetings, as a welcoming gesture that adds to their comfort.

## TRANSLATING RESEARCH TO LAY AUDIENCES

Meeting adaptations made in the early years of the VEG suggest a need to improve researchers’ ability to communicate clearly with lay audiences and develop infrastructure to ensure Veterans’ comprehension of proposed studies when providing feedback. To improve shared understanding between researchers and laypeople, a recent article on community engagement recommends the following: 1) modification of research proposals to a third to eighth-grade reading level; 2) development of a community board to review academic literature rewritten for lay audiences and summarize key points to researchers, before use in community forums; and 3) use of a trained group facilitator who is a representative community member.^15^ Additional training and funding for COE investigators to develop meaningful roles for Veteran partners throughout proposed studies could enable greater partnership.^16^

## HOMELESSNESS

VEG facilitators note Veterans’ housing status as a major barrier to sustained partnerships. Because many Veterans are recruited from VA transitional housing programs and clinical services, this population is mobile and may move far away from the meeting site. Veterans suggest that having additional correspondence with staff between meetings and technical support to join virtual meetings could support continued participation. Transportation issues, challenges navigating the campus, lack of reliable phone/Internet service, and childcare needs present additional challenges to Veteran engagement and factored into membership decline, particularly during COVID-19.^7^

## CHALLENGES TO FULL PARTNERSHIP

While the COE has resources to enable Veterans to provide guidance on a project from start to finish, the COVID-19 pandemic resulted in delays to the implementation of COE-funded pilot projects, scheduled VEG meetings, and plans to actualize coequal partnership. This limited Veterans’ ability to participate as shared leaders in all stages of the process, including research design, data collection, and analysis, and community outreach to share study findings.^3,4,16^ Plans to recruit and pair interested Veterans with related lived experience as consultants to COE-funded researchers were not fully actualized; investigator guidelines to request UCLA and VA Institutional Review Boards’ permission for Veterans to consult on data analysis or anonymized pre-publication manuscripts, as a part of their research protocols, have not yet been fully developed. Veterans also expressed the need to establish personal relationships with researchers and staff, to develop a stronger sense of trust and value when offering their unique perspectives at group proceedings. Additional training and funding for COE researchers to develop meaningful roles for Veteran partners throughout the course of proposed studies could also enable greater partnership and shared leadership in research processes.^17,18^

Because traditional advisory roles tend to be low-frequency consultation activities,^19^ further integration of Veterans into COE organizational structure and workforce could help prevent their “tokenization.”^14^ Veterans with severe psychiatric disabilities, severe substance use disorders, and homelessness histories ought to be actively recruited as research staff, early career scholars, and mid-level scholars at the COE to produce research that represents the lived experiences of stakeholders.^18,19^ While the COE has Veterans on staff, targeted recruitment of structurally vulnerable Veterans and the development of a flexible, accessible work environment have not yet been achieved.

## DISCUSSION

Although new research engagement panels with homeless-experienced Veterans are forming at the VA, little has been published about best practices when working with this population in VEGs.^11^ In the COE’s VEG, structurally vulnerable Veterans demonstrate altruism and a willingness to support research processes. This paper suggests that Veterans’ engagement throughout research projects could enable more equitable partnerships and shared leadership between researchers and Veterans. Researchers may also benefit from additional training and support to translate research to lay audiences. Lastly, Veterans may begin to set research priorities via additional community engagement strategies such as patient involvement, co-production, and community bioethics dialogues and by the development of a staff and investigator workforce that mirrors the population served.^4,17,20^ These approaches may support Veteran leadership as integral to research design and implementation. As institutional support for collaborative methodologies increases, addressing the translational and structural barriers to full Veteran partnership is crucial to the production of equitable research.

## **Abbreviations**

COE: UCLA/VA Center of Excellence on Veteran Resilience and Recovery
VAGLA: Veterans Affairs of Greater Los Angeles
VEG: Veteran Engagement Group
